# Prognostic significance of pathological response after neoadjuvant chemotherapy or chemoradiation for locally advanced cervical carcinoma

**DOI:** 10.1186/1477-7800-3-3

**Published:** 2006-02-03

**Authors:** Myrna Candelaria, José Chanona-Vilchis, Lucely Cetina, Diana Flores-Estrada, Carlos López-Graniel, Aaron González-Enciso, David Cantú, Adela Poitevin, Lesbia Rivera, Jose Hinojosa, Jaime de la Garza, Alfonso Dueñas-Gonzalez

**Affiliations:** 1 Division of Clinical Research, Instituto Nacional de Cancerología, Mexico City; 2 Pathology Department, Instituto Nacional de Cancerología, Mexico City; 3 Gynecology-Oncology Department, Instituto Nacional de Cancerología, Mexico City; 4 Division of Radiotherapy, Instituto Nacional de Cancerología, Mexico city; 5 Unidad de Investigación Biomédica en Cáncer, Instituto de Investigaciones Biomédicas, Universidad Nacional Autónoma de México, Instituto Nacional de Cancerología, Mexico City

## Abstract

**Background:**

Cisplatin-based chemoradiation is the standard of care for locally advanced cervical cancer patients; however, neoadjuvant modalities are currently being tested. Neoadjuvant studies in several tumor types have underscored the prognostic significance of pathological response for survival; however there is a paucity of studies in cervical cancer investigating this issue.

**Methods:**

Four cohorts of patients with locally advanced cervical carcinoma (stages IB2-IIIB); included prospectively in phase II protocols of either neoadjuvant chemotherapy with 1) cisplatin-gemcitabine, 2) oxaliplatin-gemcitabine, 3) carboplatin-paclitaxel or 4) chemoradiation with cisplatin or cisplatin-gemcitabine followed by radical hysterectomy were analyzed for pathological response and survival.

**Results:**

One-hundred and fifty three (86%) of the 178 patients treated within these trials, underwent radical hysterectomy and were analyzed. Overall, the mean age was 44.7 and almost two-thirds were FIGO stage IIB. Pathological response rates were as follows: Complete (pCR) in 60 cases (39.2%), Near-complete (p-Near-CR) in 24 (15.6 %) and partial (pPR) in 69 cases (45.1%). A higher proportion rate of pCR was observed in patients treated with chemoradiotherapy (with cisplatin [19/40, 47.5%]; or with cisplatin-gemcitabine [24/41, 58.5%] compared with patients receiving only chemotherapy, 6/23 (26%), 3/8 (37.5%) and 8/41 (19.5%) for cisplatin-gemcitabine, oxaliplatin-gemcitabine and carboplatin-paclitaxel respectively [p = 0.0001]). A total of 29 relapses (18.9%) were documented. The pathological response was the only factor influencing on relapse, since only 4/60 (6.6%) patients with pCR relapsed, compared with 25/93 (26.8%) patients with viable tumor, either pNear-CR or pPR (p = 0.001). Overall survival was 98.3% in patients with pCR versus 83% for patients with either pNear-CR or pPR (p = 0.009).

**Conclusion:**

Complete pathological response but no Near-complete and partial responses is associated with longer survival in cervical cancer patients treated with neoadjuvant chemotherapy or chemoradiotherapy.

## Background

Cervical cancer remains as one of the biggest killers in women around the world. The epidemiology of cervical cancer is strongly related to the standard of living of populations, thus, underdeveloped countries present the higher mortality rates which can be as high as >70 per 100 000 inhabitants and most cases are diagnosed in locally advanced disease -stages IB2-IVA- according to the FIGO classification [1].

Five randomized studies have demonstrated that survival with radiation therapy alone is lower than with radiation therapy with concomitant chemotherapy based on cisplatin [2-6]. Afterwards, a meta-analysis corroborated these findings confirming that chemoradiation offers an absolute survival benefit at 5 years of 12% [7]. Thus, cisplatin-based chemoradiation was largely accepted as the standard of care for cervical cancer patients whose treatment requires radiation. However, not only concomitant chemoradiotherapy has shown benefit in locally advanced cervical cancer; a meta-analysis of neoadjuvant chemotherapy followed by radical hysterectomy has also shown an absolute benefit of 15% at 5-year survival [8]; currently, the EORTC is conducting a randomized phase III trial comparing these two treatment modalities. On the other hand, multimodal treatments incorporating radiation, chemotherapy and surgery must be investigated in the aim to further improve the prognosis [9].

Studies in breast carcinoma, one of the tumors most frequently treated with neoadjuvant chemotherapy have underscored the importance of achieving a major or complete pathological response to prolong survival. In general, higher pathological complete response rate correlates with better survival [10,11]. On these bases it can be hypothesized that more effective neoadjuvant modalities would produce longer survival by increasing the pathological complete response rates.

There exists limited information on the value of pathological response for predicting survival in cervical cancer patients treated with neoadjuvant modalities. In particular, the meaning of pathological complete, near-complete (microscopic residual) or partial response remains unsettled. To investigate this issue, we have reviewed the outcome of the patients that underwent surgery in our series of phase II studies of neoadjuvant chemotherapy or chemoradiation followed by radical hysterectomy.

## Methods

Four phase II studies were performed on FIGO staged IB2-IIIB patients diagnosed with cervical squamous cell carcinoma, adenocarcinoma or adenosquamous carcinoma at the National Institute of Cancerology. Patients had to meet the following criteria in order to be included: 1) No previous oncological treatment; 2) older than 18 years old and younger than 70 years old; 3) functional status of 0-2 according to the WHO classification; 4) normal hematological, hepatic, renal and respiratory function according to the following parameters: hemoglobin >9 gr/L, leukocyte count >4000/mm^3^, and platelets > 100 000/mm^3^; total bilirubin and transaminases <1.5 times the high normal value; normal serum creatinine; 5) normal posteroanterior radiograph of the thorax; and 6) informed consent. The Institutional Regulatory Boards of the National Institute of Cancerology approved the protocols. Patients with history of cancer, severe systemic or uncontrolled metabolic diseases, neuropathy or psychiatric disorders were excluded.

Clinical staging was performed without anesthesia at least by two physicians of the Gynecology and Radiotherapy Departments. In the case of disagreement, the evaluation of an additional examiner was asked.

### Treatment protocols

#### Neoadjuvant cisplatin gemcitabine followed by radical hysterectomy

Treatment, toxicity and response evaluation details have been previously published [12]. Patients received 3 courses every 21 days of cisplatin at 100 mg/m^2 ^on day 1, and 1000 mg/m^2 ^of gemcitabine on days 1 and 8.

#### Neoadjuvant oxaliplatin gemcitabine followed by radical hysterectomy

Treatment, toxicity and response evaluation details have been previously published [13]. Patients received 3 courses every 21 days of oxaliplatin at 130 mg/m^2 ^on day 1, and 1250 mg/m^2 ^gemcitabine on days 1 and 8.

#### Neoadjuvant carboplatin paclitaxel followed by radical hysterectomy

Treatment, toxicity and response evaluation details have been previously published [14]. Patients received 3 courses every 21 days with carboplatin at a dose calculated according with an area under the curve (AUC) of 6, and paclitaxel at 175 mg/m^2 ^both on day 1.

#### Neoadjuvant chemoradiation with cisplatin or cisplatin gemcitabine followed by radical hysterectomy

Treatment, toxicity and response evaluation details have been previously published [15]. This was a randomized phase II study where we compared cisplatin (weekly at 40 mg/m2 for six) versus cisplatin gemcitabine (weekly at 40 mg/m2 and 125 mg/m2 respectively, both for six) concurrent to external beam radiation (50–56 Gy in 5 weeks with 2 Gy fractions) followed by radical hysterectomy.

In all these four trials, radical hysterectomy was performed within 5 weeks after the neoadjuvant treatment, if it was clinically considered that resection would be possible obtaining free surgical margins. In case of unresectable tumors, patients in trials of neoadjuvant chemotherapy received *definitive treatment *with radiation therapy (teletherapy and brachytherapy) plus 40 mg/m^2 ^weekly cisplatin for 6 applications during teletherapy. In the neoadjuvant chemoradiation trial, patients with any pathological response less than complete or near-complete received brachytherapy. In addition, in the neoadjuvant cisplatin gemcitabine no postoperative radiation was used regardless of the pathological response, whereas in the oxaliplatin gemcitabine adjuvant chemoradiation was used in all cases with a pathological response less than complete; and in the carboplatin paclitaxel, only in those cases with partial response (no chemoradiation in complete or near-complete response).

### Follow-up

Follow-up included pelvic examination every 3 months starting at treatment completion. Imaging studies, such as CT scan, were performed when considered clinically indicated.

### Evaluation of pathological response

Pathological evaluation of tumor was done by a pathologist who was blinded to the treatment received by the patient. Complete cervix was included for analysis and the response was registered as follows: Patients with no residual viable tumor cells in the surgical specimen (primary tumor and lymph nodes T0N0M0) were classified as having a pathological *complete response *(pCR); *near-complete *or microscopic response was defined with the presence of one or more foci of malignant viable cells measuring less than 1 millimeter and *partial response *when the residual tumor was larger than 1 millimeter.

### Statistical analysis

Overall survival was estimated using the Kaplan-Meier method [16] from the date of diagnosis to date of death or last follow-up. The influence of variables on survival was analyzed using the Cox regression analysis [17]. All statistical tests were two-sided, with significance defined as p < 0.05. All analysis was performed using SPSS-10 software.

## Results

### Characteristics of patients

Out of 178 patients treated within these trials, 86% (153 patients) were submitted to radical hysterectomy and are subjected to this analysis. Overall, the mean age was 44.78 (range 24–67) and almost two-thirds (91, 59.4 %) were IIB. Stage distribution according to protocol treatment is shown in table 1.

**Table 1:** Clinical stage according to neoadjuvant protocol for operated patients

Neoadjuvant protocol	Clinical stage					Total
	1B2	IIA	IIB	IIIA	IIIB	

cisplatin + gemcitabine	2	3	14	0	4	23
carboplatin + paclitaxel	7	7	22	1	4	41
oxaliplatin + gemcitabine	2	1	4	0	1	8
CT/RT with cisplatin	9	4	27	0	0	40
CT/RT with cisplatin + gemcitabine	8	9	24	0	0	41
Total	28	24	91	1	9	153

### Treatment and pathological response by group of treatment

Neoadjuvant cisplatin gemcitabine followed by radical hysterectomy. This study included 41 patients or which 23 underwent surgery. Six 6 (26%) patients had pCR and 17 pPR.

Neoadjuvant gemcitabine oxaliplatin followed by radical hysterectomy. This study included 10 patients. Only 8 of them underwent radical hysterectomy, of which three (37.5%) patient attained pCR, three (37.5%) had pNear-CR and two pPR (25%).

Neoadjuvant carboplatin paclitaxel followed by radical hysterectomy. This study included 43 patients. Of these patients, 41 (95%) underwent radical hysterectomy obtaining pCR and pNear-CR in 8 (19.5%) and 4 (9.7%) patients, respectively. The remaining 29 (70.7%) patients had pPR.

Preoperative chemoradiation with cisplatin or cisplatin gemcitabine. Eighty-three patients were included in this randomized trial. Of these 40 were in the cisplatin arm and all were operated; 43 received cisplatin and gemcitabine and 41 underwent surgery. Pathological complete, near-complete and partial responses were as follows: cisplatin arm: 19 (47.5%), 8 (20 %) and 13 (32.5%) whereas in the cisplatin gemcitabine arm rates were: 24 (58.5%), 9 (22.5%) and 8 (19.5 %) respectively.

Summarizing, pathological response rates were as follows: pCR in 60 cases (39.2 %), pNear-CR in 24 (15.6 %) and pPR in 69 cases (49.2%). A higher proportion rate of pCR was observed in patients treated with chemoradiotherapy (with cisplatin [19/40, 47.5%]; or with cisplatin-gemcitabine [24/41, 58.5%]) compared with patients receiving only chemotherapy, 6/23 (26%), 3/8 (37.5%) and 8/41 (19.5 %) for cisplatin-gemcitabine, oxaliplatin-gemcitabine and carboplatin-paclitaxel respectively (p = 0.0001), table 2.

**Table 2:** Distribution of pathological response in treatment groups

Neoadjuvant protocol	Complete response	Near-complete	Partial response	Total
cisplatin + gemcitabine	6	-	17	23
carboplatin + paclitaxel	8	4	29	41
oxaliplatin + gemcitabine	3	3	2	8
CT/RT with cisplatin	19	8	13	40
CT/RT with cisplatin + gemcitabine.	24	9	8	41
Total	60	24	69	153

### Relapse rate

A total of 29 relapses (18.9%) were documented. The analysis of relapse rate, according with the neoadjuvant treatment, and clinical stage showed no statistical significant difference among all modalities administered (p = 0.06), as well as initial clinical stage (p = 0.53). The pathological response was the only factor influencing on relapse, since only 4/60 (6.6%) patients with pCR relapsed, compared with 5/24 (20.8 %) or 20/69 (28.9 %) in patients with pNear-CR, or pPR, respectively (p = 0.001).

### Overall survival

**Figure 1:**
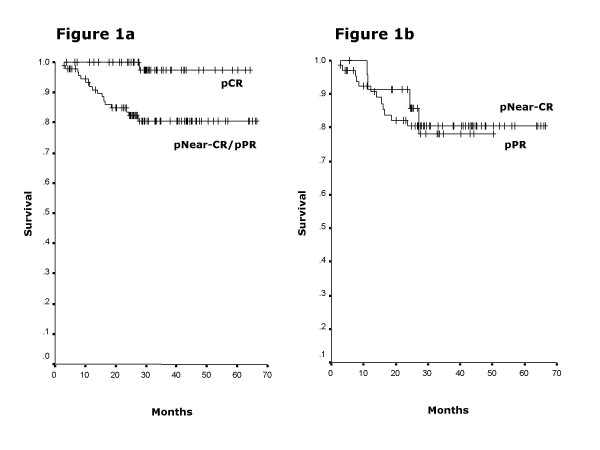
(A) Overall survival according to type of response. At a median follow-up time of 29.6 months (2.9–66.4), the survival for complete pathological response is 98.3% whereas is 83% for those with near-complete and partial response, (p = 0.009). (B) Overall survival for those with a response less than complete (near-complete and partial response). The survival for near-complete is 83.3% whereas is 82.3% for those with partial response (p = 0.820).

Survival analysis was restricted to the 153 patients that underwent radical hysterectomy. At a median follow-up time of 29.6 months (2.9–66.4) for all patients, 5-year-overall survival was 98.3% in patients with pCR versus 83% for patients with either pNear-CR or pPR (p = 0.009) (Figure 1a). Among the ones with no pCR no difference in 5-year overall survival was found between patients with pNear-CR compared with those with pPR (Figure 1b). Cox multivariate analysis was done to underscore factors influencing on survival. The only prognostic factor influencing on overall survival was pathological response (p = 0.008, 95 % CI: 1.103 -1.953). Neither the type of neoadjuvant treatment (p = 0.639, 95%CI: 0.710 -1.748), nor the initial clinical stage (p = 0.531, 95 % CI: 0.707–1.959): were factors determining the overall survival.

## Discussion

Currently, cisplatin-based chemoradiation is the standard of care for locally advanced cervical carcinoma [9]. This combined treatment has of proven efficacy even in patients treated outside of clinical trials which is certainly important as this neoplasm mainly affects socially disadvantaged women who may not comply with the treatment [18]. Nevertheless, the 5-year survival of locally advanced cervical cancer patients is around 70% hence other therapeutic approaches must be tested in order to further improve prognosis. Among these, neoadjuvant chemotherapy and neoadjuvant chemoradiation are being tested in phase III randomized trials.

A number of studies of neoadjuvant chemotherapy or concurrent chemoradiation in several tumor types underscore the importance of achieving a major or complete pathological response to prolong survival. Although it has been argued that patients with no residual carcinoma have a better outcome because of selection bias rather than the effects of preoperative therapy, if a high pathological complete response rate correlates with better survival then it can be hypothesized that more effective neoadjuvant modalities would produce longer survival by increasing the pathological complete response rates.

There are some indications that at least for cervical cancer this may hold true. Chang et al., [19] performed a randomized trial in IB bulky patients of neoadjuvant chemotherapy using the "quick scheme" of cisplatin 50 mg/m^2 ^day 1, bleomycin 25 mg/m^2 ^days 1–3, and vincristine 1 mg/m^2 ^day 1, repeated every 10 days for three courses against pelvic radiotherapy alone. They found no differences in survival which can be the result of the low rate (4.6%) of pathological complete response rate. Other phase II trials using these "old regimens" report similar low rates of pathological responses; such as 6.6% by Singh et al., [20], 9.5% by Porzio [21], 7% by Kim [22], and 12% by Benedetti-Panici et al., [23]. On the contrary, with newer regimens of chemotherapy incorporating drugs such as gemcitabine [12,13], vinorelbine [24], paclitaxel [14,25] or irinotecan [26], complete pathological response rates are higher which may lead potentially to better survival rates. In this sense, we have reported in a comparison of two consecutive phase II trials similar survival of neoadjuvant chemotherapy with cisplatin gemcitabine followed by surgery in which complete pathological response was 26% versus chemoradiation with cisplatin in locally advanced cervical carcinomas [27], but better survival with that neoadjuvant treatment than radiation alone [28]. Pending confirmation from the randomized phase III trial that is ongoing in the EORTC it is very likely that neoadjuvant chemotherapy followed by surgery at best would be equivalent that the current standard of chemoradiation. These data strongly suggest that neoadjuvant chemotherapy trials need to be even more effective to show superiority over standard chemoradiation. However, so far there are no indications of such superiority with current drugs or combinations. For instance, a phase III trial of neoadjuvant chemotherapy in cervical cancer comparing a doublet -ifosfamide and cisplatin versus a triplet -ifosfamide, cisplatin and paclitaxel found no better results on survival by the incorporation of a third drug [29]. These results have led investigators to assay neoadjuvant chemoradiation instead of only chemotherapy to improve pathological responses and survival.

Several prospective phase II trials of preoperative chemoradiation with cisplatin and 5-fluorouracil have been performed. Resbeut et al., reported a complete pathological response rate of 40% in 40 patients staged from IB to IVA [30]; Jurado et al., reported a complete pathological response rate of 67.5% in 40 patients [31] while the complete pathological response rate was 54.2% in 25 patients staged as IIB-IIIA in a third study [32]. Interestingly, the disease-free and overall survival these trials reported is very encouraging further supporting the hypothesis than prognosis can be improved by highly effective neoadjuvant therapies. In line with that, we performed a randomized phase II trial comparing cisplatin versus cisplatin and gemcitabine as sensitizers to radiation. Our results demonstrate that pathological complete responses can be indeed increased from 47.5% with cisplatin to 58.5% by adding gemcitabine, a powerful radiosensitizer to cisplatin [15].

There exists limited information on the value of pathological response for predicting survival in cervical cancer patients treated with neoadjuvant modalities. In particular, the meaning of pathological complete, near-complete (microscopic residual) or partial response remains unsettled. The results of this analysis shed some light on this issue. We found that for cervical cancer a complete pathological response by either neoadjuvant chemotherapy or neoadjuvant chemoradiation is indicative of very favorable survival whereas we found no different prognosis for those with no complete response irrespective of the "amount" of residual disease either microscopic (near-complete response) or minor or not change in disease (partial response). Resbeut et al., [30] made no distinction between complete and microscopic (near-complete) responses, instead they reported only complete or partial pathological response, nor integrated pathological response to their survival analysis, however; only one of the 16 patients with complete response had local recurrence suggesting its association with good prognosis. Jurado et al., defined complete pathological response as tumor eradication higher than 95% and found a 9-year local control rate of 100% versus 78% (p = 0.004) and overall survival of 93% versus 70% (p = 0.038) for complete and partial responders respectively [31]. Finally, Mancuso's study registered complete, near-complete (as microscopic residual) and partial (as macroscopic residual) and reported no local failures among the 13 patients with complete response suggesting that complete responders have the most favorable prognosis [31,32]. The prognostic significance of pathological complete response is also suggested in the setting of pre-exenterative chemotherapy for recurrent cervical cancer patients. In such modality, only one of four patients with pathological complete response and two out of four with residual disease relapsed [33].

The discrepancy in the prognostic significance of complete versus near-complete also called as major response or microscopic residual is also observed in other tumors. In rectal and non-small cell lung treated with chemoradiation [34,35] and bladder cancer treated with chemotherapy [36], complete and near-complete were predictive of better survival whereas in breast cancer the better prognosis is not observed for any response less than complete [37-39]. Whether the different prognostic meaning of pathological responses result from technical issues such as the number of patients and follow-up in studies, methods for analyzing and reporting the surgical specimens or result from the type of neoadjuvant treatment or even the type of tumor needs to be further studied. It is important however, to emphasize that among the studies on cervical cancer, the present report is the one reporting the higher number of patients and under two different neoadjuvant treatments which strengths our results on the prognostic significance of complete but no near-complete or partial pathological response.

## Conclusion

Complete pathological response but no near-complete and partial responses are associated with very favorable survival in cervical cancer patients treated with neoadjuvant chemotherapy or chemoradiotherapy. These data strongly suggest that every effort must be made to obtain a high pathological complete response rate in order to improve survival. The value of adjuvant therapies for patients with a pathological response less than complete must be investigated in this subset of locally advanced cervical cancer patients treated under neoadjuvant protocols.

## Competing interests

The author(s) declare that they have no competing interests.

## Authors' contributions

MC conceived the study and analyzed the data and wrote the manuscript; J C-V did the pathological evaluation; LC was in charge of the chemotherapeutic management; AP, LR and JH performed the radiation management; DF-E participated in compilation of information and data management; CL-G, AG-E and DC participated in the surgical management of patients; AD-G conceived the study and wrote the manuscript. All authors participated in the discussion and critically read the manuscript.
